# Evaluation of survival of patients with locally advanced head and neck cancer treated in a single center

**DOI:** 10.1016/j.bjorl.2019.06.006

**Published:** 2019-07-23

**Authors:** Fred Muller dos Santos, Gustavo Arruda Viani, Juliana Fernandes Pavoni

**Affiliations:** a Universidade de São Paulo (USP), Faculdade de Filosofia, Ciências e Letras, Departamento de Física, Ribeirão Preto, SP, Brazil; b Universidade de São Paulo (USP), Faculdade de Medicina de Ribeirão Preto (FMRP), Ribeirão Preto, SP, Brazil

**Keywords:** Head and neck, Surgery, Radiotherapy, Chemoradiation, Survival

## Abstract

**Introduction:**

Even with improved treatment outcomes with multimodality approaches, the question of what is the best initial treatment for locally advanced head and neck cancer still remains unanswered.

**Objective:**

To review the overall survival of a large cohort of head and neck cancer, patients with locally advanced head and neck cancer treated in a single institution.

**Material and methods:**

We studied a cohort of patients with locally advanced head and neck cancer treated in our institution in the last fifteen years. To gather a large sample of patients with adequate follow-up time, a cross-check between ours and Fundação Oncocentro de São Paulo databases were done. We included patients with head and neck cancer, clinical or pathological staging III or IV, treated with surgery followed by radiotherapy or surgery plus chemoradiation or radiotherapy alone or chemoradiation alone.

**Results:**

796 patients with locally advanced head and neck cancer were included, 88% male, 44% age >60 years and 76% stage IV. The tumor location was the oral cavity (34%), oropharynx (27%), hypopharynx (17%) and larynx (17%). The treatment groups were chemoradiation alone (39.7%), surgery plus chemoradiation (26.3%), surgery followed by radiotherapy (18.5%) and radiotherapy alone (15.5%). Comparing the clinical variables between the treatment groups significant differences in age and clinical stage were observed. With a median follow up of 7.5 years (1–16 years), for the entire cohort, the overall survival at 5 and 10 years was 34.8% and 28%. The overall survival at 5 and 10 years was 16.7% and 12.2% for radiotherapy alone, 38.8% and 26.3% for surgery followed by radiotherapy, 28% and 16.6% for chemoradiation alone, and 37.3% and 23.2% for surgery plus chemoradiation. The staging IV (*p* = 0.03) and radiotherapy alone (*p* = 0.05), had a worst survival in multivariate analysis. Surgical groups vs. chemoradiation alone had no significant difference for overall survival.

**Conclusion:**

The present study is the largest cohort of locally advanced head and neck cancer of Brazilian patients to evaluate treatment outcomes. Although there were significant clinical differences between surgical and radiotherapy groups, surgery or chemoradiation alone as the initial treatment resulted in no significant difference in survival.

## Introduction

Head and neck cancers account for 3% of all cancers in the United States.^1^, ^2^ According to data from the Brazilian National Cancer Institute (INCA), in 2017 there were approximately 23,000 new cases of head and neck cancer.^3^ Despite improving treatment outcomes with multimodality treatment for the locally advanced disease, there is still a high rate of death from the disease.^2^, ^4^, ^5^, ^6^, ^7^ The treatment options for locally advanced disease traditionally includes surgery followed by adjuvant radiotherapy (S + RT) or adjuvant chemoradiation (S + CRT), or only CRT.^2^ The use of chemotherapy combined with radiation in both clinical scenarios (adjuvant or exclusive) has increased since the publication of randomized clinical trials in the last several decades.^5^, ^6^, ^7^ In general, these studies revealed a significant benefit for locoregional control, disease-free survival and overall survival with an increased rate of acute and late toxicity.^5^, ^6^, ^7^, ^8^, ^9^ In the landmark trial conducted by the Radiation Therapy Oncology Group (RTOG) an improved in the overall survival rate with CRT vs. RT alone (*p* = 0.001) was observed, with a median follow-up of 10 years.^8^ Another problem evolving the management of LAHNC is the absence of well-designed studies comparing head to head surgery vs. CRT as upfront treatment.^10^, ^11^

Consequently, even with the improvement in the treatment outcomes with the multimodality approaches, the question whether CRT produces comparable results to the surgical treatment with an extended follow-up remains controversial.^10^ The scarcity of data of good quality comparing these different treatment modalities for LAHNC has resulted in varied choices for the upfront treatment.^12^, ^13^, ^14^ In clinical practice, radiation oncologists and surgeons are daily put required to decide what is the best treatment alternative in terms of survival for patients with LAHNC.^12^

The decision about the selection for primary treatment requires a multidisciplinary team.^12^ The main driving factors considering the decision are the primary tumor site, disease extension, age, comorbidity, patient’s preference and morbidity of each treatment approach.^15^ The choice of initial therapy should also consider the experience and technology available at the medical institution.^16^

In clinical scenarios where there is no randomized clinical trial to guide the decision making in clinical practice, data from large databases with a long follow-up time can add a clarity to the clinical issues. However, there is limited evidence from developing countries describing the treatment outcomes of LAHNC.

In Brazil, there is a national cancer registry database maintained by the federal government. This national database is linked to smaller regional databases. Our institution is included in the FOSP foundation database,^17^ one of these regional databases. Thus, based on the identified registries in the last 15 years of Head and neck cancer treated in our institution and the FOSP database,^17^ we design a cohort study to evaluate the survival of patients with LAHNC treated in a single institution of a developing country.

## Methods

This cohort study was designed to evaluate the overall survival of patients with LAHNC treated in a single institution. To gather a large sample of Head and Neck Cancer (HNC) patients with adequate follow-up time, we include patients treated in the last fifteen years in our institution. To guarantee an adequate follow-up time, we cross-checked our database with the database from FOSP.^17^ This institution is linked to the State Department of Health, which receives cancer data from hospitals in the state of São Paulo through regional centers called Cancer Hospital Registries. It configures a cohort of secondary data, since the individuals diagnosed and treated at the hospital are evaluated for the rest of their life through annual follow-ups, after identifying and collecting information in the medical records.

### Ethical standards

The FOSP website is a public domain with the objective of allowing the health professionals the possibility of performing tabulations and specific analyzes, according to their interest and needs.^18^ Therefore, the present study did not need approval from the local ethics committee. All data obtained for this article came from the intersection between the patients treated at our institution and patients registered in the FOSP public database.^17^

### Analysis process

The information was filtered to limit the treatment period from 2000 to 2014, and Head and Neck Cancer cases that refer to ICDs “C00 to C14” and “C30 to C32” according to the 10th International Statistical Classification of Diseases and Related Health Problems (ICD-10). We included only patients with histological diagnostic of cancer from head and neck with a clinical or pathological staging III or IV treated with surgery followed by adjuvant treatment or treated for radiotherapy or chemoradiation alone. Regarding the radiotherapy schedule, only conventional fractionation was included. To reduce the influence of radiotherapy dose, only cases were included with doses higher than 60 Gy for adjuvant radiotherapy and doses higher than 69.8 Gy for radiotherapy alone or combined with chemotherapy. During the long period, different radiation techniques were employed, therefore, conventional, conformational and intensity modulated radiotherapy techniques were included. We excluded patients with metastatic disease (clinical Stage IVC), or patients with early disease (clinical Stage I‒II), patients treated with induction chemotherapy, or chemotherapy without cisplatin or patients treated with hyper fractionation or hypofractionation radiotherapy.

The analyses performed were based on the information contained in the database such as age, sex, the morphology of the disease, topography, clinical stage, treatments performed, follow-up time and death. The cases were categorized by the initial treatment type employed: radiotherapy alone (RT); adjuvant radiotherapy (S + RT); adjuvant chemoradiation (S + CRT); and chemoradiation (CRT).

Patients submitted to radiotherapy in the period between 2000 and 2008 were treated using conventional treatment technique (2DRT), with doses varying from 60 Gy to 70 Gy. Patients submitted to surgery receive between 60 and 66 Gy depending on the pathological findings, and patients treated with RT or CRT alone doses varied between 69.2 Gy to 70.9 Gy. Since 2009, conformational radiotherapy (3DRT) and IMRT treatments were applied to most of the patients with the same doses. All patients were treated by a linear accelerator or a cobalt machine. The radiotherapy treatment volumes followed the treatment guidelines according to the site of the disease current at the period. The chemotherapy was based on cisplatin each to three weeks with 100 mg/m^2^ in the majority of the cases, and when necessary weekly cisplatin with 30 mg/m^2^.

### Statistical analysis

The survival curves were created using the R Commander and Survival packages of the software R, which consists of a free programming environment for statistical and graphical computation. The survival analysis was counted from the end of radiotherapy treatment until death or last patient information. The survival analysis was performed by the Kaplan–Meier estimator method. The Cox stepwise method was used for the multivariate analysis. The Hazard Ratio (HR) was calculated with the regression model that allows the evaluation of the independent variables as well as their relevance in a set of other parameters. The recognized prognostic factors were included in the multivariate analysis. Age (≥60 or <60 years), sex (male or female), clinical stage (Stage IV or Stage III), upfront treatment (RT, CRT, S + RT, S + CRT) and tumor location (oral cavity, oropharynx, hypopharynx, larynx, nasopharynx and maxillary sinus). A *p*-value < 0.05 and 95% CI was considered statistically significant.

## Results

In our database during the period between 2000 and 2015 around 7000 patients received radiotherapy treatment. The cross-check of our database with the FOSP database resulted in 6115 patients treated with radiotherapy with a follow-up of more than 1 year. Regarding the HNC patients, in general, we identified 990 with the diagnosis of HNC and 796 patients with LAHNC (clinical Stage III and IV), and a follow-up with more than one year.

**Figure 1:**
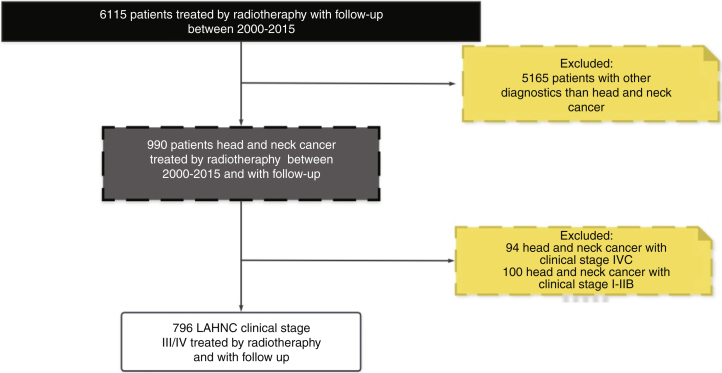
Flowchart of patients included in the study.

**Figure 2:**
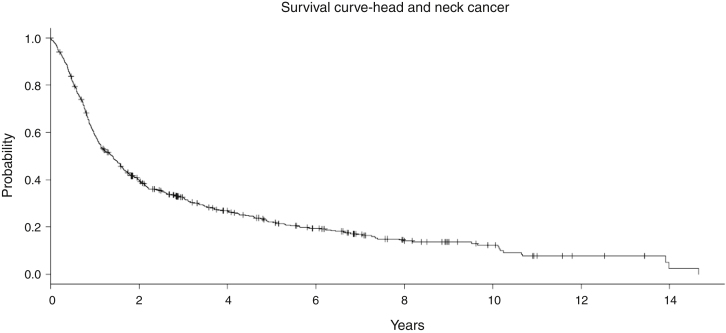
Patients characteristics according to the tumor site and gender.

Therefore, the present study included 796 patients with LAHNC treated in the last fifteen year in our institution. Fig. 1 describes the reasons and the number of patients excluded from the study according to our inclusion criterion. In the entire period, the most common treatment employed was CRT (39.7%), S + CRT (26.3%), S + RT (18.5%) and RT (15.5%). During the period, there was an increase in the indications for RT, CRT and S + CRT, as described in Table 1. In general, the most common anatomical sites were the oral cavity (34%), oropharynx (27%), hypopharynx (17%) and larynx (17%) (Fig. 2).

**Table 1:** Patient and tumor characteristics according to the treatment types.

	RT alone		S + RT		CRT		S + CRT	
	n	%	n	%	n	%	n	%
Age								
<60	51	41.5	80	54.4	197	62.3	135	64.3
≥60	72	58.5	67	45.6	119	37.7	75	35.7
Gender								
Male	104	84.6	124	84.4	291	92.1	185	88.1
Female	19	15.4	23	15.6	25	7.9	25	11.9
Stage								
III	25	20.3	46	31.3	54	17.1	54	25.7
IV	98	79.7	101	68.7	262	82.9	156	74.3
Period								
2000‒2004	28	27.2	42	31.6	51	19.0	33	18.6
2005‒2009	27	26.2	57	42.9	74	27.6	63	35.6
2010‒2014	48	46.6	34	25.6	143	53.4	81	45.8
Site								
Oral cavity	39	31.7	63	42.9	97	30.7	73	34.8
Oropharynx	43	35.0	19	12.9	107	33.9	44	21.0
Hipopharynx	18	14.6	19	12.9	57	18.0	44	21.0
Nasopharynx	7	5.7	1	0.7	26	8.2	3	1.4
Larynx	16	13.0	45	30.6	29	9.2	46	21.9
Histology								
Squamous cell carcinoma	117	95.1	140	95.2	308	97.5	202	96.2
Others	6	4.9	7	4.8	8	2.5	8	3.8
Follow-up time for treatment (years)	7.6		7.2		6.1		7.4

The most frequent histological subtype in all groups was Squamous Cell Carcinoma (SCC) (96%). Evaluating the distribution of clinical variables among the treatment groups there was a significant difference with more patients with clinical Stage IV in both RT groups, and younger patients and clinical Stage (III) in surgical groups (*p* < 0.05). Regarding the tumor site, a significant difference was observed with oropharynx more often in the RT and the CRT groups, while the larynx was more frequent in the surgical groups (*p* < 0.05).

**Figure 3:**
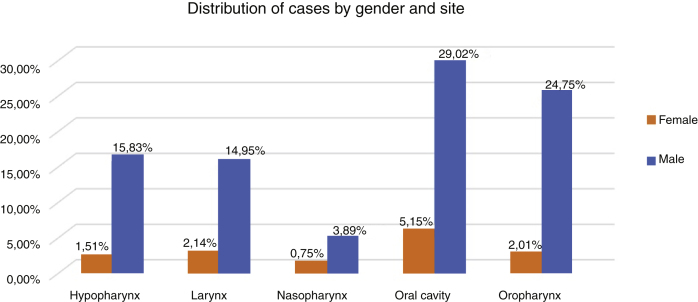
Overall survival of the entire cohort of patients with locally advanced head and neck independently of the treatment modality employed.

In the entire cohort, the median follow-up was 7.5 years (1–18 years). No significant difference in the follow-up time was observed among the treatment groups (Table 1). In general, the Overall Survival (OS) at 5 years was 34.8% and at 10 years it was 29.9% (Fig. 3). In the RT group with 123 patients, the OS at 5 and 10 years was 16.7% and 12.2%, respectively. For S + RT group, the OS was 38.8% at 5 years and 26.3% at 10 years. For the CRT group, the OS was 28% at 5 years and 16% at 10 years. Finally, S + CRT had a rate of OS at 5 years of 37% and 23% at 10 years, respectively.

In the multivariate analysis (Table 2), the age and sex parameters did not present significance, respectively. Patients with clinical stage IV had poor survival, with *p* = 0.045 and Hazard Ratio (HR) 1.386 (95% CI 1.1–1.9). Regarding the treatment group, RT alone showed the worst OS rate, followed by CRT (HR = 2.298 and <0.0001), S + RT (HR = 3.220 and <0.0001) and S + CRT (HR = 3.650, *p* < 0.0001). When the RT group was excluded, and we compared only the three groups (S + RT vs. S + CRT vs. CRT), no significant difference was observed. Finally, concerning to the tumor site, there was a lower OS rate for the hypopharynx, followed by oropharynx, oral cavity, nasopharynx, and larynx, with a tendency to be significant (Table 2).

**Table 2:** Multivariate cox regression analysis for overall survival.

	n	%	HR	95% CI	*p*-Value
Age (≥60)				Lower–upper	
<60	463	58.2	1.144	0.867–1.509	0.343
≥60	333	41.8			
Gender (male)					
Male	704	88.4	1.517	0.932–2.468	0.093
Female	92	11.6			
Stage (Stage 4)					
III	179	22.5	1.386		0.045
IV	617	77.5			
Treatment (RT)					
RT	123	15.5	–	–	‒
S + RT	147	18.5	3.220	2.111–4.909	<0.0001
CRT	316	39.7	2.298	1.549–3.409	<0.0001
S + CRT	210	26.4	3.650	2.366–5.627	<0.0001
Tumor site (hypopharynx)					
Hypopharynx	138	17.3	–	–	–
Oral cavity	272	34.2	1.050	0.719–1.531	0.802
Oropharynx	213	26.8	1.035	0.691–1.552	0.866
Sinus maxillary	37	4.6	1.395	0.488–3.987	0.534
Larynx	136	17.1	1.598	0.959–2.664	0.072

## Discussion

Over the last decades, there has been a shift in the treatment strategy for LAHNC. During this period, the number of patients undergoing radiotherapy and chemotherapy has increased, and the number of those treated with surgery alone has decreased.^8^, ^19^, ^20^

The shifting in the management of LAHNC has been supported since the publication of an individual patient meta-analysis.^7^ In 2000, Pignol et al. identified 63 trials (10,741 patients) comparing locoregional treatment with or without chemotherapy. The combined treatment yielded a pooled hazard ratio of death of 0.90 (95% CI 0.85‒0.94, *p* < 0.0001), corresponding to an absolute survival benefit of 4% at 2 and 5 years in favor of chemotherapy.^7^

Currently, several guidelines including the American Society of Clinical Oncology (ASCO) consider the combined treatment appropriate for most patients with T3 and T4 tumors without invasion into soft tissues through the cartilage.^7^

The management of LAHNC is a significant clinical problem with a poor 5 year survival rate not exceeding 50%, despite the multimodality treatment.^9^ Many studies have shown that medical expertise, guideline-based treatment, high-volume facilities, multidisciplinary support, infrastructure availability, and patient’s socioeconomic status can affect therapeutic outcomes.^15^, ^16^, ^21^ There is a lack of studies providing information on patients from developing countries with LAHNC treated with the available resources. Therefore, our objective in performing this cohort study was to evaluate the treatment outcome concerning overall survival in a large cohort of Brazilian patients treated at a public service with a long follow-up to identify any differences employing surgical (S + RT or S + CRT) or organ-preserving approaches (CRT or RT).

The outcomes observed in our cohort confirm this dismal survival with a long-term follow-up. In general, the 5 and 10 year survival rate was about 35% and 18%, independently of the treatment modality chosen as upfront treatment (Fig. 3). It is difficult to compare this result with randomized trials, mainly due to the inclusion of many patients with multiple anatomical sites and Stage IV (unresectable disease). However, if we look at the treatment outcomes of studies including unresectable disease, our results mirror the outcomes in the literature. For instance, Aldenstein et al. conducted a randomized trial testing the addition of chemotherapy to radiotherapy in patients with unresectable LAHNC.^9^ Patients were randomized to a single daily fractionated radiation (70 Gy at 2 Gy/d), or identical radiation therapy with concurrent bolus cisplatin, given on days 1, 22 and 43. Between 1992 and 1999, 295 patients were randomized. With a median follow-up of 41 months, the 3 year OS for RT was 23%, compared with 37% for CRT B (*p* = 0.014).^9^

Although this study includes an extended period, in our institution we have followed the NCCN guidelines. In Brazil, the NCCN is the most frequently used (70%),^22^ thus during the period, patients with intermediate risk for local recurrence, i.e., perineural invasion, lymph-vascular invasion, and T3/T4 received adjuvant RT. Patients with the presence of positive margins, extracapsular extension or positive lymph node received adjuvant CRT. The results of surgical groups were similar to other studies reported in the literature. In a multicenter retrospective study including 176 patients with larynx cancer, 65 (37%) with clinical Stage III, 51 were submitted to organ-preserving treatment, and 14 underwent laryngectomy.^10^ Of the 111 patients with clinical Stage IV, 42 were treated with non-surgical treatment and 69 underwent total laryngectomy. Overall survival at 3 years was 58% for Stage III and 42% for Stage IV. The choice of treatment did not influence the survival for Stages III (*p* = 0.56) and IV (*p* = 0.93). However, there was a trend towards better outcomes with surgical treatment, especially in patients with advanced disease.^10^

Despite the incorporation of the concept of multimodality treatment, there is still considerable discussion regarding the optimal treatment strategies for patients with LAHNC.^12^, ^17^, ^20^ Primary surgery has experienced a recent revival with the advent of newer surgical techniques and a better knowledge of tumor biology.^21^ Although CRT in the last decades has increased its indication, some authors have questioned the organ preservation and survival of these patients with a long follow-up.^23^ Evaluating our data, the survival achieved by CRT is comparable to the surgical group, independent of the adjuvant treatment employed. However, with a long follow-up (over 5 years), our data showed a tendency to lower survival in CRT. For us, this tendency is related to a more advanced age and stage in the CRT group.

Consequently, when the treatment groups were placed in the multivariate analysis by the Cox estimator, there was a better performance of the surgical intervention followed by CRT and the worst performance for survival in the RT alone group.

The CRT and RT group suffered from selection bias with more advanced clinical stage (IV) when compared with surgical groups. The selection bias is natural for LAHNC, once these tumors are unresectable or patients are of advanced age or without adequate clinical condition to support the surgical procedure being, therefore, better treated by CRT or RT. For patients who are selected to non-surgical treatment, our data makes clear the necessity for a careful selection of patients to receive the combined treatment. The requirement of incorporating chemotherapy to radiotherapy schedule for this kind of patient is demonstrated by the profound difference in survival at five and ten years of about 2.5 times, with an absolute difference of 10%. These results reinforce the need for multidisciplinary discussion to guarantee better overall survival for these patients.

Our study has some caveats: first, this is a retrospective study of patients treated in a single institution. To gather a large sample of LAHNC patients with long follow-up, we combined our data with follow-up data from FOSP. Due to this, unfortunately, we did not get data about toxicity, local control and salvage treatment of those patients. Nevertheless, we believe that in general, this work represents the outcomes of LAHNC treated in structured cancer hospitals in Brazil.

## Conclusion

The present study is the largest cohort of LAHNC of Brazilian patients published to evaluate the difference in treatment outcomes. Although there were significant clinical differences between surgical and radiotherapy groups such as age, clinical stage, and tumor sites, our results show no significant differences for survival between surgical treatments as initial treatment compared to CRT. However, RT alone showed the worst survival compared to other treatment options. This data call attention to the multidisciplinary discussion to adequately select patients to the CRT. The selection is also critical for patients with clinical Stage IV and tumors from hypopharynx sites once these variables had the worst survival in multivariate analysis. In general, the treatment outcomes from a cohort of Brazilian patients were comparable to survival rates of randomized trials including unresectable disease. Further studies should be conducted to create useful quality indicators for properly selecting patients with LAHNC for different treatment options.

## Conflicts of interest

The authors declare no conflicts of interest.
